# The Effect of Nano-Epigallocatechin-Gallate on Oxidative Stress and Matrix Metalloproteinases in Experimental Diabetes Mellitus

**DOI:** 10.3390/antiox9020172

**Published:** 2020-02-20

**Authors:** Adriana Elena Bulboaca, Paul-Mihai Boarescu, Alina Silvia Porfire, Gabriela Dogaru, Cristina Barbalata, Madalina Valeanu, Constantin Munteanu, Ruxandra Mioara Râjnoveanu, Cristina Ariadna Nicula, Ioana Cristina Stanescu

**Affiliations:** 1 Department of Pathophysiology, Iuliu Haţieganu University of Medicine and Pharmacy Cluj-Napoca, Victor Babeş Street, no. 2-4, 400012 Cluj-Napoca, Romania; 2 Department of Pharmaceutical Technology and Biopharmaceutics, Iuliu Haţieganu University of Medicine and Pharmacy Cluj-Napoca, Victor Babeş Street, no. 41, 400012 Cluj-Napoca, Romania; 3 Department of Physical Medicine and Rehabilitation, Iuliu Haţieganu University of Medicine and Pharmacy Cluj-Napoca, Viilor Street, no. 46-50, 400347 Cluj-Napoca, Romania; 4 Department of Medical Informatics and Biostatistics, Iuliu Haţieganu University of Medicine and Pharmacy Cluj-Napoca, Louis Pasteur Street, no. 6, 400349 Cluj-Napoca, Romania; 5 Department of Medical Rehabilitation, “BagdasarArseni” Emergency Clinical Hospital Bucharest, Berceni Street, no. 12, 041915 Cluj-Napoca, Romania; 6 Department of Pneumology, Iuliu Haţieganu University of Medicine and Pharmacy Cluj-Napoca, B.P. Hasdeu Street, no. 6, 400371 Cluj-Napoca, Romania; 7 Department of Ophthalmology, Iuliu Haţieganu University of Medicine and Pharmacy Cluj-Napoca, Clinicilor Street, no. 3-5, 400006 Cluj-Napoca, Romania; 8 Department of Neurology, Iuliu Haţieganu University of Medicine and Pharmacy Cluj-Napoca, Victor Babeş Street, no. 43, 400012 Cluj-Napoca, Romania

**Keywords:** epigallocatechin-gallate, liposomes, diabetes mellitus, oxidative stress

## Abstract

Background: The antioxidant properties of epigallocatechin-gallate (EGCG), a green tea compound, have been already studied in various diseases. Improving the bioavailability of EGCG by nanoformulation may contribute to a more effective treatment of diabetes mellitus (DM) metabolic consequences and vascular complications. The aim of this study was to test the comparative effect of liposomal EGCG with EGCG solution in experimental DM induced by streptozotocin (STZ) in rats. Method: 28 Wistar-Bratislava rats were randomly divided into four groups (7 animals/group): group 1—control group, with intraperitoneal (i.p.) administration of 1 mL saline solution (C); group 2—STZ administration by i.p. route (60 mg/100 g body weight, bw) (STZ); group 3—STZ administration as before + i.p. administration of EGCG solution (EGCG), 2.5 mg/100 g b.w. as pretreatment; group 4—STZ administration as before + i.p. administration of liposomal EGCG, 2.5 mg/100 g b.w. (L-EGCG). The comparative effects of EGCG and L-EGCG were studied on: (i) oxidative stress parameters such as malondialdehyde (MDA), indirect nitric oxide (NOx) synthesis, and total oxidative status (TOS); (ii) antioxidant status assessed by total antioxidant capacity of plasma (TAC), thiols, and catalase; (iii) matrix-metalloproteinase-2 (MMP-2) and -9 (MMP-9). Results: L-EGCG has a better efficiency regarding the improvement of oxidative stress parameters (highly statistically significant with *p*-values < 0.001 for MDA, NOx, and TOS) and for antioxidant capacity of plasma (highly significant *p* < 0.001 for thiols and significant for catalase and TAC with *p* < 0.05). MMP-2 and -9 were also significantly reduced in the L-EGCG-treated group compared with the EGCG group (*p* < 0.001). Conclusions: the liposomal nanoformulation of EGCG may serve as an adjuvant therapy in DM due to its unique modulatory effect on oxidative stress/antioxidant biomarkers and MMP-2 and -9.

## 1. Introduction

Consuming green tea has been linked to human health and longevity for centuries. In particular, green tea catechins are involved in many biological processes such as antioxidant activity and modulation of various cellular lipid and protein metabolisms [1]. Green tea contains a great amount of polyphenols (flavonols, flavones, and flavanols) with similar structure, possessing lots of therapeutic active components including catechin, epicatechin, epicatechin-3-gallate, and epigallocatechin-3-gallate (EGCG) [2]. EGCG is the most active and abundant compound (65% of total catechin content) [3,4].

Green tea therapeutic effects have been studied intensively, proving beneficial in various diseases such as cancer [5], hyperlipidemia [6,7], cardiovascular diseases [8,9], neurodegenerative diseases [10,11], and infectious diseases [12,13]. Some reports also suggest that daily consumption of tea catechins may help in controlling type 1 [14] and type 2 diabetes mellitus [1]. It has been demonstrated that green tea consumption reduces fasting glucose levels, an effect mediated by EGCG [15]. Lipophilic EGCG has been shown to reduce glycemia and serum lipids in experimental diabetes mellitus induced by streptozotocin (STZ) in rats [16].

Type 1 diabetes mellitus (DM) is associated with an autoimmune-mediated destruction of pancreatic beta cells, leading to absolute insulin deficiency [17]. One of the most used experimental models for testing various therapies addressing type 1 DM is based on STZ administration. STZ induces type 1 DM, with destruction of pancreatic beta cells and associated insulin deficiency, as a result of its cytotoxic effect, mediated by increased synthesis of reactive oxygen species (ROS) and subsequent inflammation [18,19,20]. A protective effect of EGCG on pancreatic beta cells has been already demonstrated in experimental studies [21]; meanwhile, oral chronic administration of EGCG proved to have hypoglycemic and hypolipidemic effects and to reduce oxidative stress in streptozotocin-diabetic rats [22]. EGCG can exert antioxidant, anti-inflammatory, antiangiogenetic, and antifibrotic effects [2]. The catechol or galloyl groups from catechins act as scavengers for metal ions, reducing further production of free radicals [23]. Another essential effect is represented by the scavenging activity for free radicals, through phenoxyl compounds [24]. EGCG treatment can also reduce oxidative stress by increasing the level of antioxidant enzymes, such as superoxide dismutase (SOD), glutathione peroxidase (GP), and catalase (CAT), emerging in an antiapoptotic consequence [25].

Matrix metalloproteinases (MMPs) are a family of enzymes (peptidases) involved in degradation and remodeling of extracellular matrix (ECM) [26]. Recent studies reveal that MMPs can regulate chemokines and cytokines synthesis, thus participating in innate immunity processes, inflammation, and angiogenesis [27]. MMPs can be generated by various cell types, such as endothelial cells and mononuclear cells of the immune system [28]. Pathological induction of MMP synthesis is associated with an imbalance between synthesis and degradation of ECM proteins leading to ECM degradation [29]. High glucose ambience influences the MMPs’ increased synthesis and low tissue inhibitors of MMPs (TIMP) activity [30]. Increased levels of MMP-2 and MMP-9 are observed in type 1 diabetic patients and animal models, such as STZ-induced diabetes mellitus in rats [31,32], and are associated with microvascular complications of DM [28]. 

Analyzing the EGCG therapeutic properties and pharmacokinetic parameters, considerable individual differences and variations between results were noted [33]. EGCG is highly lipophilic, which explains its low bioavailability (0.2% to 2% of the total load ingested by healthy people), mainly because a large amount of the ingested EGCG is degraded by local microbiota and does not enter into the blood circulation [34]. Improvement of bioavailability and stability of EGCG can be obtained by encapsulation in nanoparticles [35]. Catechin nanoemulsions proved to be stable for long periods of time (120 days at 4 °C) [36]. Liposomes, assembled from phospholipid bilayers similar to cell membranes, are one of the nanoparticles frequently used for drug delivery [23]. Their biphasic character makes them suitable for being carriers for both hydrophilic (in the central aqueous compartment) and hydrophobic (in lipid bilayers) compounds [37,38]. Nanoformulation by encapsulation in liposomes could also facilitate the solubility for hydrophobic particles [4]. Through all of these properties, liposomes can offer an enhanced bioavailability, stability, and shelf life for sensitive ingredients [39].

The aim of this study was to investigate the effect of two forms of EGCG (EGCG solution and liposomal EGCG) on oxidative stress parameters, antioxidant capacity, serum MMP-2 and -9, and pancreatic and liver function in STZ-induced diabetes mellitus in rats.

## 2. Materials and Methods 

### 2.1. Materials

The substances used for liposomal preparation were: Epigallocatechin-gallate (EGCG) derived from green tea (Sigma-Aldrich, Steinheim, Germany); 1,2-dipalmitoyl-sn-glycero-3-phosphocholine (DPPC): *N*-(carbonyl-methoxypolyethylenglycol-2000)-1,2-distearoylsn-glycero-3-phosphoethanolamine Na-salt (MPEG-2000-DSPE) (Lipoid GmbH, Ludwigshafen am Rhein, Germany); and cholesterol (CHO) obtained from sheep wool (Sigma-Aldrich, Steinheim, Germany). All other solvents and reactive substances were obtained from Sigma-Aldrich, Steinheim, Germany, and had an analytical degree of purity. 

### 2.2. Experimental Model

The study was approved by the Ethic Committee of the University and by the National Sanitary Veterinary Authority number 137/13.11.2018. Twenty-eight male Wistar-Bratislava rats were procured from the Centre of Experimental Medicine, University of Medicine and Pharmacy, Cluj-Napoca, Romania. The rats weighed 200–250 g, were kept in polypropylene cages, with day–night regimen, at constant temperature (24 ± 2 °C) and humidity (60 ± 5%). Free access to food (standardized pellets from Cantacuzino Institute, Bucharest, Romania) and water was provided to all animals. The animals were randomly divided into 4 groups (7 rats/group). The groups were organized as follows: 

group 1—control group (C)—with intraperitoneal (i.p.) administration of 1 mL saline solution, 

group 2—STZ administration by i.p. route (STZ), 

group 3—STZ administration as before + i.p. administration of EGCG solution (EGCG), 

group 4—STZ administration as before + i.p. administration of liposomal EGCG (L-EGCG). 

Each medication was dissolved in saline solution (0.9% sodium chloride) and the volume administrated i.p. was 1 mL [19]. The following doses were used: STZ—60 mg/100 g body weight (b.w.) [40]; EGCG in saline solution or in liposomal form were freshly prepared and were administrated i.p. in a dose of 2.5 mg/100 g b.w./day as pretreatment, two consecutive days before STZ administration [41]. Intraperitoneal administration was preferred as a method that improves EGCG bioavailability, compared to low bioavailability with oral administration [42].

Blood samples were taken at 48 h after STZ administration, under ketamine anesthesia (5 mg/100 g bw, i.p. route) from retro-orbital sinus, followed by rat euthanasia by cervical dislocation [43]. Rats with glucose higher or equal to 200 mg/dL were considered to have diabetes mellitus [20].

### 2.3. Preparation and Physicochemical Characterization of EGCG-Loaded Liposomes

For the preparation of liposomes, we used a modified film hydration method [44,45]. The lipid double-layer components, having a 70 mM concentration (DPPC:MPEG-2000-DSPE:CHO = 4.75:0.25:1 molar ratio), were dissolved in ethanol in a round-bottomed glass flask. Ethanol was evaporated at 45 °C under low pressure; the lipid film product was hydrated with a solution of EGCG diluted in highly purified water, pH = 5.00, at the same temperature. The resulted liposomal dispersion was then extruded through polycarbonate membranes with 200 nm final pore dimension, with LiposoFastLF-50 equipment (Avestin Europe GmbH, Mannheim, Germany). Unencapsulated EGCG particles were removed by dialysis method, using Slide-A-Lyzer filters (cassettes) with 10 kDa molecular weight cut-off. 

To assess the amount of liposomal-loaded EGCG, we used a spectrophotometric method—the reaction with Folin–Ciocâlteu reagent (Merck, Darmstadt, Germany) [46]. During this procedure, a dilution of liposomal dispersion with methanol 1:10 (*v*/*v*) was made, and a UV-VIS spectrophotometer (Specord 200 Plus, Analytik Jena, Überlingen, Germany) measured the absorbance value. 

The size and polydispersity index of liposomes were assessed by dynamic light scattering method (with a 90° scattering angle), and the zeta potential was measured by laser Doppler electrophoresis; a Zetasizer Nano ZS analyzer was used for both assessments (Malvern Instruments Co., Malvern, UK). 

The mean liposomal concentration of the L-EGCG solution was about 900 μg/mL, and encapsulation efficiency was over 80%. Liposomal vesicles’ mean size was 170 nm, and polydispersity index was less than 0.2, meaning that the vesicles’ size and uniformity were appropriate to ensure a prolonged circulation in the blood. Aggregative stability was ensured by values of 51.83 mV of the zeta potential.

### 2.4. Oxidative Stress and Antioxidant Parameters Assessment

Parameters associated with oxidative stress and antioxidant status were determined from collected blood samples. The parameters used to assess oxidative stress were: malondialdehyde (MDA) [47], indirect nitric oxide (NOx) synthesis assessment [48], and total oxidative status (TOS) [49]. Antioxidant status parameters were represented by total antioxidant capacity of plasma (TAC) [50], thiols [51], and catalase [52]. All measurements were performed using a Jasco V-350 UV-VIS spectrophotometer (Jasco International Co, Ltd., Tokyo, Japan). Matrix metalloproteinases (MMPs) were appraised from serum using a rat ELISA kit (Boster Biological technology, Pleasanton, CA, USA) and a Stat Fax 303 ELISA reader (Quantikine, McKinley Place NE, MN, USA).

### 2.5. Assessment of Beta Pancreatic Cells and Hepatic Cells Function

Glycemia was measured at 48 h after DM induction, as it was previously observed that STZ induces significant beta cell death at 48 after administration [53]. Glycemia was also used as a parameter for pancreatic function changes induced by experimental diabetes mellitus. Hepatic cytolysis was assessed by serum levels of aspartate aminotransferase (AST) and alanine aminotransferase (ALT) measured by a standardized technique (Vita Lab Flexor E, Spankeren, The Netherlands) [40]. 

### 2.6. Data Analysis

The SPSS software package version 21.0 (SPSS Inc., Chicago, IL, USA) was used for statistical analysis and graphic representations. The acceptable error threshold was *p* = 0.05. In order to describe the continuous quantitative data, we used the arithmetic mean and the standard deviation (SD). The distribution of investigated markers in groups was plotted as individual values (circles) and median (line), as recommended by Weissgerber and coauthors [54]. The Kruskal–Wallis ANOVA was used to test the differences in the investigated markers. The Mann–Whitney test was used in post hoc analysis when significant differences were identified by the Kruskal–Wallis ANOVA test. 

## 3. Results

No rat died during the experiment, so the analysis was conducted on all seven rats in each group. All P values for comparison between groups are presented in Supplementary Table S1.

In our experimental model, diabetes mellitus was successfully induced by STZ: all rats that received STZ were definitely diabetic, proven by glycemia >200 mg/dL and values significantly higher in diabetic rats compared to control group: 401.81(11.31) mg/dL versus 84.27 (2.87) mg/dL, respectively (expressed as mean and standard deviation), with a *p*-value < 0.001. Also, hepatic damage was detected in the STZ group, quantified by significant elevation of transaminases AST and ALT (Table 1). 

**Figure 1:**
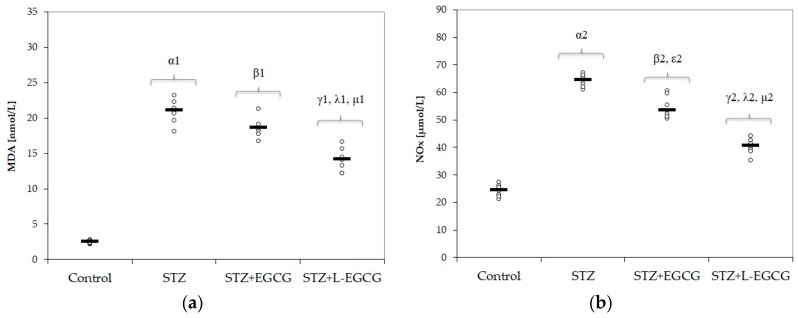

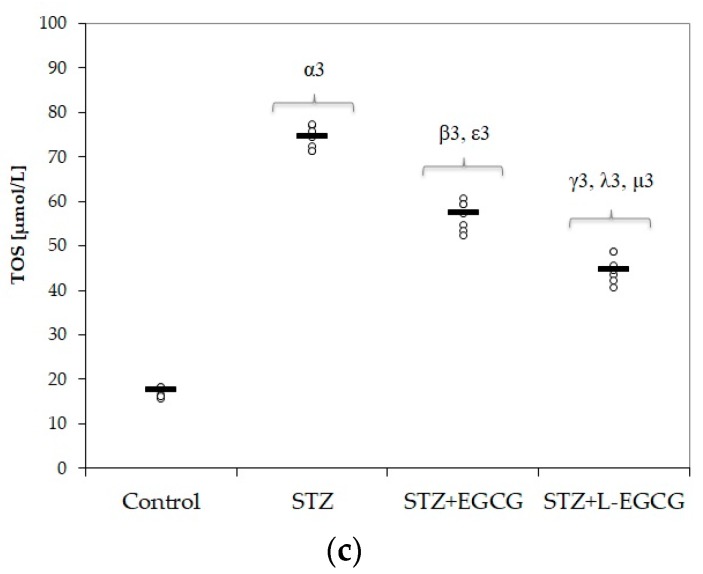
Distribution of oxidative stress intensity by groups: (**a**) MDA (malondialdehyde), (**b**) NOx (indirect nitric oxide), (**c**) TOS (total oxidative status) on all study groups (7 rats/group). STZ = streptozotocin control; STZ + EGCG = STZ and EGCG solution i.p. as pretreatment; STZ + L-EGCG = STZ and liposomal EGCG i.p. as pretreatment. The symbol–number codes correspond to the *p*-values < 0.05 as follows: α—STZ compared to control; β—STZ + EGCG compared to control; ε—STZ + EGCG compared to STZ; γ—STZ + L-EGCG compared to control; λ—STZ + L-EGCG compared to STZ; µ—STZ + L-EGCG compared to STZ + EGCG.

**Figure 2:**
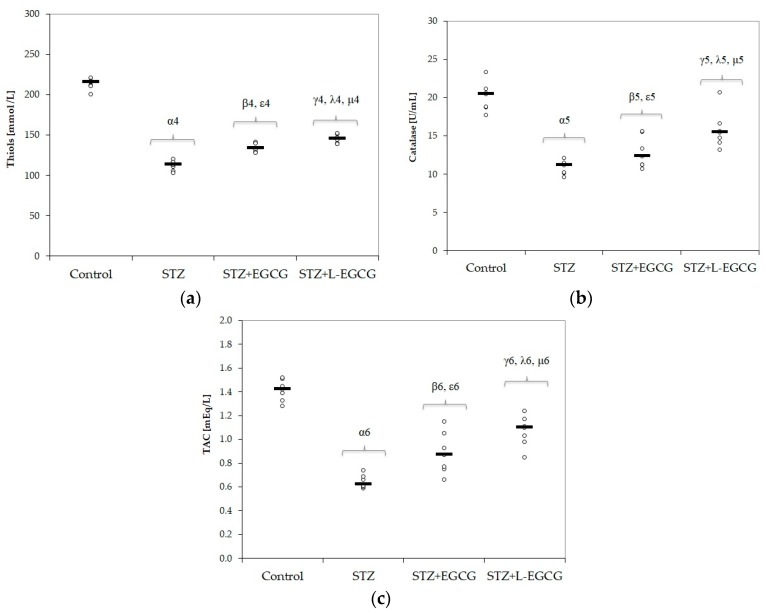
Distribution of plasmatic antioxidant capacity by groups: (**a**) Thiols, (**b**) Catalase, (**c**) TAC (total antioxidant capacity) on all study groups (7 rats/group). STZ = streptozotocin control; STZ + EGCG = STZ and EGCG solution i.p. as pretreatment; STZ + L-EGCG = STZ and liposomal EGCG i.p. as pretreatment. The symbol–number codes correspond to the *p*-values < 0.05 as follows: α—STZ compared to control; β—STZ + EGCG compared to control; ε—STZ + EGCG compared to STZ; γ—STZ + L-EGCG compared to control; λ—STZ + L-EGCG compared to STZ; µ—STZ + L-EGCG compared to STZ + EGCG.

Oxidative stress parameters (MDA, NOx, and TOS) significantly increased after induction of DM (*p*-values <0.001 in all items, Figure 1a–c, Table 1). MMP-2 and MMP-9 levels were significantly higher in the STZ-induced DM group compared with control group (*p*-values <0.001, Figure 4a,b, Table 1). Serum antioxidant capacity, measured by thiol, catalase, and TAC levels, was significantly reduced in diabetic rats compared to control animals (*p*-values < 0.001 in all items, Figure 2a–c, Table 1).

In the diabetic group pretreated with EGCG, oxidative stress parameters NOx and TOS were significantly reduced compared to the untreated STZ group (with *p*-values of 0.017 and <0.001, respectively, Figure 1b,c).

All antioxidant parameters (thiols, catalase, and TAC) were significantly higher in the STZ-treated group (*p*-values of < 0.001, 0.026, and 0.017 respectively, Figure 2a–c). 

**Figure 3:**
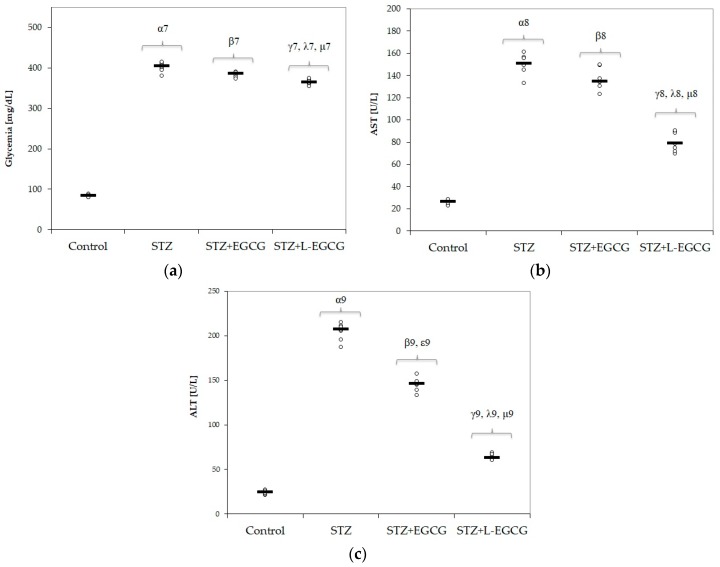
Distribution of (**a**) Glycemia, (**b**) AST (aspartate aminotransferase), (**c**) ALT (alanine aminotransferase) on all study groups (7 rats/group). STZ = streptozotocin control; STZ + EGCG = STZ and EGCG solution i.p. as pretreatment; STZ + L-EGCG = STZ and liposomal EGCG i.p. as pretreatment. The symbol–number codes correspond to the *p*-values < 0.05 as follows: α—STZ compared to control; β—STZ + EGCG compared to control; ε—STZ + EGCG compared to STZ; γ—STZ + L-EGCG compared to control; λ—STZ + L-EGCG compared to STZ; µ—STZ + L-EGCG compared to STZ + EGCG.

**Figure 4:**
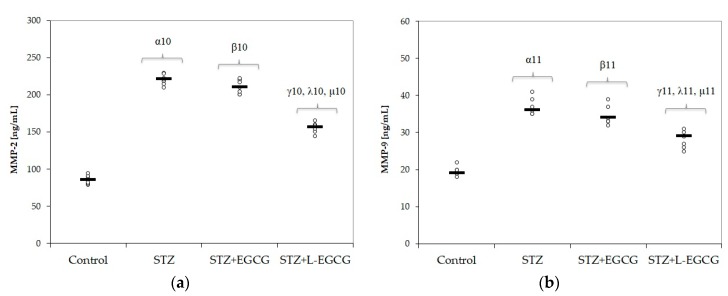
Distribution of matrix metalloproteinase (MMP): (**a**) MMP-2 and (**b**) MMP-9 on all study groups (7 rats/group). STZ = streptozotocin control; STZ + EGCG = STZ and EGCG solution i.p. as pretreatment; STZ + L-EGCG = STZ and liposomal EGCG i.p. as pretreatment. The symbol–number codes correspond to the *p*-values < 0.05 as follows: α—STZ compared to control; β—STZ + EGCG compared to control; γ—STZ + L-EGCG compared to control; λ—STZ + L-EGCG compared to STZ; µ—STZ + L-EGCG compared to STZ + EGCG.

No significant differences were noted in MDA and MMP values between the pretreated group with EGCG compared to the untreated STZ group (Figure 1a, Figure 4a,b). Also, glycemia and liver parameters were not significantly different in the EGCG pretreated group, with the exception of a decrease in ALT (*p*-value = 0.038, Figure 3c). 

In the STZ group pretreated with L-EGCG, all oxidative stress parameters were significantly decreased and serum antioxidant capacity parameters were all increased, with better results compared to the STZ group pretreated with EGCG (*p* < 0.017, Figure 1 and Figure 2). Also, the L-EGCG solution improved glycemic values and decreased transaminases levels better than EGCG (*p* < 0.001, Figure 3). The MMP levels were significantly lower in the L-EGCG-treated group compared to the diabetic untreated group or compared to the STZ group pretreated with EGCG (<0.001, Figure 4).

The Kruskal–Wallis ANOVA test identified significant differences between the groups with diabetes and EGCG pretreatment for all evaluated parameters (*p*-values < 0.0001). The post hoc analysis identified significant differences in most of the cases with better protection for the EGCG-treated group, and significantly higher protection when liposomal EGCG solution was used (Figure 1, Figure 2, Figure 3 and Figure 4).

## 4. Discussion

### 4.1. Protective Effects of EGCG on Pancreatic and Hepatic Cell Function in Diabetic Rats

In our study, EGCG reduced blood glucose levels in pretreated animals but the reduction was not statically significant (Table 1, Figure 3). Some of the antidiabetic effects of EGCG are suggested to be the suppression of appetite, adjustment of dietary fat emulsification in the gastrointestinal tract, inhibition of gastrointestinal lipolysis, and reduction of nutrient absorption enzymes [55]. The most significant hypoglycemia was obtained in liposomal EGCG-pretreated groups. This indicates a protective effect of EGCG on pancreatic cell function. Meng et al. showed that EGCG can inhibit inflammation by reducing reactive oxygen species and downregulating the production of inducible nitric oxide synthetase (iNOS) [56]. Furthermore, EGCG increases glucose tolerance [57] and decrease HbA1c levels in STZ-induced diabetes in rats, contributing to further prevention of diabetic complications [58]. Another suggested mechanism of EGCG’s protective effect is the increased glucose uptake due to promoting the glucose transporter-4 (GLUT4) translocation in skeletal muscle, through activation of both phosphoinositol 3-kinase and AMP-activated protein kinase pathways [58]. EGCG also increases tyrosine phosphorylation of insulin receptors, having an insulin-like effect on H4IIE hepatoma cell lines [59]. 

The liver is extremely adversely affected in type 1 diabetes mellitus. In our study, we found elevated AST and ALT levels, showing liver damage, in STZ diabetic rats (Table 1, Figure 3). In STZ-induced diabetes, transaminases elevation is the consequence of the toxic effect of STZ on hepatocytes, which induces lipid peroxidation, oxidative stress enhancement, peroxisome proliferation, and mitochondrial dysfunction [60,61,62]. Rodriguez et al. identified increased NO levels and hepatic oxidative stress in STZ-induced diabetic rats [63]. In our study, pretreatment with EGCG decreased ALT levels, preventing hepatic damage induced by STZ. Furthermore, liposomal EGCG administration significantly reduced AST and ALT values, confirming the enhanced protective effect of L-EGCG on hepatic cells. Other studies also demonstrated the hepatic-protective effect of green tea extracts in hepatic injury reflected by decreased serum transaminase levels, and improved structural changes in histopathological examination [64]. Moreover, long-time consumption of EGCG (in healthy Wistar rats) decreases age-induced hepatic damage by lowering the ALT and AST serum levels and improving microscopic changes of the liver tissue due to the aging process [65]. 

### 4.2. Effect of EGCG on Oxidative Stress Parameters and Plasmatic Antioxidant Capacity

In this study, increased levels of MDA, NO, and TOS were observed in diabetic rats (Table 1 and Figure 1), together with low levels of antioxidant biomarkers such as thiols, catalase, and TOS (Table 1 and Figure 2). Pretreatment with EGCG and L-EGCG induced protection against STZ toxic effects, as demonstrated by reduction of oxidative stress parameters (Table 1, Figure 1) and by enhancement of antioxidant defense (Table 1, Figure 2), with best results for the liposomal form. STZ-induced diabetes in experimental models is followed by an enhanced production of reactive oxygen species (ROS) and consumption of cell antioxidant systems, as a consequence of necrotic and apoptotic degeneration of pancreatic β cells [66,67]. Hyperglycemia itself is another factor generating intracellular ROS [68]. Oxidative stress (by excessive ROS production, auto-oxidation of glycated proteins, and increased lipid peroxidation) and decreased antioxidant capacity (free radical scavengers and enzymatic systems) are also involved in the pathogenesis of diabetic complications [69,70,71,72]. 

Green tea component EGCG is a flavonoid with antioxidant and anti-inflammatory properties conferred by its particular structure, a flavanol core and two gallocatechol rings, which are able to bind metal ions and scavenge free oxygen radicals. As a consequence, EGCG exerts direct antioxidant effects (scavenger of ROS and cheater of metal ions), but also indirect antioxidant effects (inductor of antioxidant enzymes, such as catalase, and inhibitor of oxydases, such as NADPH—nicotinamide adenine dinucleotide phosphate, lipoxygenase, or xantin-oxydase) [73]. Anti-inflammatory effects of EGCG were also related to the increase of circulating levels of interleukin-10 (an anti-inflammatory cytokine) in nonobese diabetic mice [14]. EGCG can decrease lipid peroxidation in the liver, kidney, and brain, and reduce lymphocyte DNA damage in diabetic mice [74].

EGCG has low bioavailability which can be modified by incorporation in special drug delivery systems. Because of its highly lipophilic nature, EGCG is suitable for incorporation in liposome nanoparticles, composed of phospholipid bilayers. Minnelli et al. showed that pretreatment of adult retinal pigmented epithelium (ARPE) cells with EGCG encapsulated in magnesium liposomes increases the survival of cells exposed to hydrogen peroxide (H_2_O_2_), with better preserved mitochondria structure on electron microscopy examination, showing the superior antioxidant activity of L-EGCG compared with free EGCG [75]. In this regard, natural antioxidant products could be a promising therapeutic option for prevention of diabetes mellitus and its complications, conferring protection against oxidative damage by liposomal nanostructure encapsulation [69]. 

### 4.3. EGCG Effect on Matrix Metalloproteinases

In the present study, serum levels of MMP-2 and -9 increased after DM induction and were better modulated by L-EGCG (Table 1 and Figure 4). In experimental models of DM, increased MMP-2 expression and activity were linked to elevated ROS levels and oxidative stress, with consecutive pancreatic beta cell apoptosis, showing MMP-2′s important role in DM pathogenesis [76]. Thus, inhibition of intracellular MMP-2 expression is an essential target for beta cell protection and DM prevention. There is also a postulated connection between MMP production and inflammatory process and proinflammatory cytokine production associated with DM. Chemokines such as MCP-1 and NF-kB can induce MMP overproduction in DM [77]. After their secretion as inactive forms, proinflammatory molecules contribute to further transformation of MMPs in active forms by different proteases that are implicated in their cleavage [38]. MMPs are also involved in regulation and duration of immune response, endothelial cell function, vascular smooth muscle migration and proliferation, Ca^2+^ signaling pathways, and vessel contraction, all of these consistently influencing vascular remodeling in DM [78,79]. 

Activated inflammatory cells such as leucocytes can contribute to endothelial cell dysfunction and vascular damage by direct and indirect pathways. Indirect loops comprise augmentation of MMP production by proinflammatory cytokines synthesized in activated leucocytes [70].

Activation of MMP-2 and MMP-9 is important in pathogenesis of diabetic microangiopathic complications such as diabetic retinopathy, nephropathy, and neuropathy [39]. Diabetic retinopathy, by inducing apoptosis of retinal endothelial cells and by degrading the junction proteins, is followed by increased vascular permeability [80,81]. In experimental models of DM, increased oxidative stress activates MMP-2, and antioxidant therapies inhibit the development of diabetic retinopathy by modulating retinal MMP-2 levels [32,82]. Diabetic nephropathy, one of the most severe microangiopathy in diabetes mellitus, is also characterized by MMP overexpression and accelerated ECM degradation, both being a hallmark of associated histopathologic changes [30]. MMPs’ increased synthesis can also lead to neuronal injury through blood–nerve barrier (BNB) disruption, contributing to the neuropathic pain associated with diabetic neuropathy [83,84].

The multiple and complex roles exhibited by MMPs are explained by their multiple localizations. MMP-2 and MMP-9 are colocalized in vessel walls and atherosclerotic plaque, being involved in endothelial dysfunction and DM macrovascular complication and vascular remodeling [85,86]. Wang et al. reported a protective effect of EGCG after i.p. administration, by reducing the plasma levels of TNF-α, IL-6, and monocyte chemoattractant protein-1 (MCP-1) [38]. There is also evidence that EGCG can inhibit MMP-2 activation [87]. Multiple compounds of green tea can inhibit MMP-2 and -9, but the most efficient ones proved to be EGCG and epigallocatechin (EGC) [88]. Therefore, we chose the EGCG compound for our experimental study. Moreover, liposomal encapsulation brings an increased bioavailability with better results in reducing oxidative stress biomarkers and MMP plasma level. EGCG reduces MMP-2 activity by targeting the fibronectin type II repeated regions 1 and 3 of MMP-2, binds the amino acids that constitute the exosite of this enzyme, and hinders proper positioning of the substrate [89]. Due to its antioxidants effects and inhibitory action on the protein tyrosine kinases, EGCG reduces MMP-9 activity by reducing its release from the activated neutrophils [90].

From our knowledge, this is the first experimental study addressing liposomal EGCG effects in experimental DM induced by STZ in rats. Decreasing the hepatic and pancreatic damage due to STZ administration is a valuable effect of liposomal EGCG. 

### 4.4. Potential Limitations of the Study

No measurements of EGCG and L-EGCG in the blood or pancreatic and hepatic tissue were done in this study since such quantifications were outside of our aim. Future studies could be conducted to measure the concentration of EGCG and L-EGCG in the blood and tissues. Moreover, oxidative stress parameters and MMPs could be measured in liver and pancreas tissue. Another limitation of our study is that the evaluation of endogenous insulin levels and measurement of HOMA-IR for endogenous pancreatic function were not performed.

Future studies should also investigate the effects of long-term administration of EGCG and L-EGCG on DM and its complications, as this study was focused on assessing their effects 48 h after DM induction.

## 5. Conclusions

L-EGCG pretreatment reduces oxidative stress biomarkers and MMP plasma levels 48 h after DM induction. Further studies are needed to detect other particularities regarding the EGCG protective mechanisms in order to improve their therapeutic efficiency. Due to the beneficial effects of EGCG nanoformulation proven by this study on oxidative stress, antioxidative defense, and MMP-2 and -9, we propose that L-EGCG could be considered as a novel adjuvant therapy in DM management.

## Figures and Tables

**Table 1:** Values of oxidative stress parameters, antioxidants levels, glycemia, hepatic enzymes, and matrix metalloproteinases in the four groups, expressed as mean and standard deviation.

Parameter	Control (*n* = 7)	STZ (*n* = 7)	STZ + EGCG (*n* = 7)	STZ + L-EGCG (*n* = 7)
MDA [nmol/mL]	2.52(0.24)	20.94(1.67)	19.83(1.1)	14.1(1.67)
NOx [μmol/L]	24.35(2.24)	64.34(2.26)	60.63(2.65)	40.36(2.89)
TOS [μmol/L]	17.19(1.05)	74.22(2.63)	66.68(3.45)	44.84(3.06)
Thiols [mmol /L]	213.4(6.64)	112.33(6.02)	131.1(3.17)	145.64(5.14)
Catalase [U/mL]	20.12(1.87)	10.87(0.87)	12.81(1.69)	15.8(2.42)
TAC [mEq/L]	1.41(0.09)	0.64(0.06)	0.83(0.14)	1.07(0.13)
Glycemia [mg/dL]	84.27(2.87)	401.81(11.31)	391.1(10.55)	365.3(6.56)
AST [U/L]	26.03(2.16)	150.37(9.16)	141.5(9.45)	80.67(8.88)
ALT [U/L]	24.63(2.25)	204.58(9.8)	193.17(6.57)	64.18(3.42)
MMP-2 [ng/mL]	86.14(5.96)	221(7.19)	217.71(7.23)	156(6.73)
MMP-9 [ng/mL]	19.57(1.27)	37(2.24)	36.29(2.56)	28.14(2.19)

MDA = malondialdehyde; NOx = indirect nitric oxide; TOS = total oxidative status; AST= aspartate aminotransferase; ALT = alanine aminotransferase; MMP-2 = matrix metalloproteinase 2; MMP-9 = matrix metalloproteinase 9; STZ = streptozotocin control; STZ + EGCG = STZ and EGCG solution i.p. as pretreatment; STZ + L-EGCG = STZ and liposomal EGCG i.p. as pretreatment.

## Supplementary Materials

The following is available online at https://www.mdpi.com/2076-3921/9/2/172/s1, Table S1: *p*-values for comparisons between the study groups for all studied parameters.
