# Supplementary material for: The Effect of Nano-Epigallocatechin-Gallate on Oxidative Stress and Matrix Metalloproteinases in Experimental Diabetes Mellitus
Source: Antioxidants (Basel). 2020 Feb 20;9(2):172. doi: 10.3390/antiox9020172 (PMC7070619; doi:10.3390/antiox9020172)
Supplement: Supplementary file 1 [file antioxidants-09-00172-s001.pdf]

The effect of nano-epigallocathehingallate on oxidative stress and matrix metalloproteinases in experimental diabetes mellitus

Adriana Elena Bulboaca, Paul-Mihai Boarescu*, Alina Silvia Porfire*, Gabriela Dogaru*, Cristina Barbalata, Madalina Valeanu, Constantin Munteanu, Ruxandra Mioara Râjnoveanu, Cristina Ariadna Nicula and Ioana Cristina Stanescu.

Supplementary Table 1. P-values for comparisons between the study groups for all studied parameters

Parameter	Number	STZ compared to Control α	STZ + EGCG compared to Control β	STZ + EGCG compared to STZ ε	STZ + L-EGCG compared to Control γ	STZ + L-EGCG compared to STZ λ	STZ + L-EGCG compared to STZ + EGCG: μ
MDA [nmol/mL]	1	<0.001	<0.001	0.128	<0.001	<0.001	<0.001
NOx [μmol/L]	2	<0.001	<0.001	0.017	<0.001	<0.001	<0.001
TOS [μmol/L]	3	<0.001	<0.001	<0.001	<0.001	<0.001	<0.001
Thiols [mmol /L]	4	<0.001	<0.001	<0.001	<0.001	<0.001	<0.001
Catalase [U/mL]	5	<0.001	<0.001	0.026	0.015	<0.001	0.017
TAC [mEq/L]	6	<0.001	<0.001	0.017	<0.001	<0.001	0.011
Glycaemia [mg/dL]	7	<0.001	<0.001	0.097	<0.001	<0.001	<0.001
AST [U/L]	8	<0.001	<0.001	0.128	<0.001	<0.001	<0.001
ALT [U/L]	9	<0.001	<0.001	0.038	<0.001	<0.001	<0.001
MMP-2 [ng/mL]	10	<0.001	<0.001	0.098	<0.001	<0.001	<0.001
MMP-9 [ng/mL]	11	<0.001	<0.001	0.117	<0.001	<0.001	<0.001
